# Improved weed segmentation in UAV imagery of sorghum fields with a combined deblurring segmentation model

**DOI:** 10.1186/s13007-023-01060-8

**Published:** 2023-08-22

**Authors:** Nikita Genze, Maximilian Wirth, Christian Schreiner, Raymond Ajekwe, Michael Grieb, Dominik G. Grimm

**Affiliations:** 1 Technical University of Munich, TUM Campus Straubing for Biotechnology and Sustainability, Bioinformatics, Schulgasse 22, 94315 Straubing, Germany; 2 Weihenstephan-Triesdorf University of Applied Sciences, Bioinformatics, Petersgasse 18, 94315 Straubing, Germany; 3 Technical University of Munich, TUM School of Computation, Information and Technology (CIT), Boltzmannstr. 3, 85748 Garching, Germany; 4 Technology and Support Centre in the Centre of Excellence for Renewable Resources (TFZ), Schulgasse 18, 94315 Straubing, Germany

**Keywords:** Weed detection, Segmentation, Machine learning, Computer vision, Deblurring, UAV

## Abstract

**Background:**

Efficient and site-specific weed management is a critical step in many agricultural tasks. Image captures from drones and modern machine learning based computer vision methods can be used to assess weed infestation in agricultural fields more efficiently. However, the image quality of the captures can be affected by several factors, including motion blur. Image captures can be blurred because the drone moves during the image capturing process, e.g. due to wind pressure or camera settings. These influences complicate the annotation of training and test samples and can also lead to reduced predictive power in segmentation and classification tasks.

**Results:**

In this study, we propose DeBlurWeedSeg, a combined deblurring and segmentation model for weed and crop segmentation in motion blurred images. For this purpose, we first collected a new dataset of matching sharp and naturally blurred image pairs of real sorghum and weed plants from drone images of the same agricultural field. The data was used to train and evaluate the performance of DeBlurWeedSeg on both sharp and blurred images of a hold-out test-set. We show that DeBlurWeedSeg outperforms a standard segmentation model that does not include an integrated deblurring step, with a relative improvement of $13.4 \%$ in terms of the Sørensen-Dice coefficient.

**Conclusion:**

Our combined deblurring and segmentation model DeBlurWeedSeg is able to accurately segment weeds from sorghum and background, in both sharp as well as motion blurred drone captures. This has high practical implications, as lower error rates in weed and crop segmentation could lead to better weed control, e.g. when using robots for mechanical weed removal.

**Supplementary Information:**

The online version contains supplementary material available at 10.1186/s13007-023-01060-8.

## Background

Weed control in agricultural fields is a time-sensitive and critical task. Depending on the quantity and distribution of weeds in agricultural fields, farmers must consider different strategic and economic options, ranging from chemical to mechanical or manual weed control. These decisions depend on several factors, including efficacy, cost, and regulations. The most common way to control weeds is with herbicides, which can have a negative impact on groundwater quality and thus cause public concern [1]. Mechanical weed control is a possible solution to deal with the resulting environmental degradation, although there are challenges such as efficiency, management and erosion effects. Therefore, automated image analysis combined with Unmanned Aerial Vehicle (UAV) imagery is a fast and effective method to reliably detect weeds in agricultural landscapes [2–7]. Automatic weed detection is difficult due to many factors, such as large variations in plant species, occlusions, or changing outdoor conditions. Therefore, modern deep learning based techniques have shown promising results in many agricultural tasks and are replacing conventional methods due to higher accuracy and flexibility [8–11]. In addition, UAVs come with their own difficulties, such as degraded image quality due to challenging illumination conditions on agricultural sites. In addition, the image quality of these images is prone to motion blur, as either the UAV or also possibly the plants may move due to wind pressure during the capture [12]. In addition, the drone and camera settings can affect the influence of motion blur due to the interdependence of flight speed and shutter speed.

Although motion blur is common when using UAVs to capture images in agricultural fields, its effect on the predictive power of weed segmentation models has not been widely studied. In our previous work, we focused on weed segmentation in motion blurred UAV captures [12]. The captures were degraded by different levels of motion blur, and we concluded that it is possible to train deep learning-based segmentation models on motion blurred captures. However, the annotation process of these degraded captures is more difficult and, more importantly, highly time consuming. The weed segmentation model from our recent study [12] consists of a feature extractor and a semantic segmentation architecture to decode the features. Here, residual networks [13] serve as feature extractors because they mitigate the vanishing gradient problem using identity skip connections and allow data to be passed from any layer directly to any subsequent layer. For our semantic segmentation architecture, we used UNet [14], which was originally developed for biomedical tasks. It has also been shown in a variety of non-medical domains that this architecture can achieve sufficient segmentation results even when little training data is available [15–17]. UNet uses skip connections between the encoder and decoder to link information from the encoder and decoder layers.

In general, computer vision tasks such as object detection or segmentation are often degraded by motion blur [18, 19], making motion deblurring an important task in image enhancement. Motion blur is a common form of image distortion. It depends on the magnitude of several overlapping effects [20] and can be described mathematically with respect to different sources of blur. However, most deblurring approaches are based on the simplified blur model [21–24], which is described by the following equation:

1 $$\begin{aligned} b = H \cdot s+n. \end{aligned}$$

It follows that a blurred image *b* is the result of a convolution between the underlying sharp image *s* and the blur kernel *H* and an addition of noise *n*. Deblurring tasks can be divided into two categories: non-blind deblurring if the blur kernel is known, and blind deblurring otherwise. In addition, the spatial invariance of the blur kernel is often assumed to produce uniform blur [25]. However, in the agricultural domain, wind-induced shifts are not only possible for the drone (i.e., camera shake), but might also occur at the plant level. Therefore, these real-world images are often degraded by spatially varying and blind blur. Blind blur removal is a highly ill-posed inverse problem, as there are many possible outcomes of a deblurred image [26, 27]. Deep learning methods for image deblurring [28, 29] typically do not explicitly estimate the underlying blur kernel, but by using a Convolutional Neural Network (CNN), relevant image features are extracted and used to directly restore a sharp output image. Therefore, these models are trained on blurry-sharp image pairs. There are several types of deblurring methods already existing in the research community, ranging from encoder-decoder-based models [30–32] to transformer-based models [33–35] to generative models [36–38].

Recently, Chen et al. proposed a computationally efficient encoder-decoder model called NAFNet [39]. The authors used a single-stage UNet architecture with skip connections and concluded that nonlinear activation functions are not necessary for image deblurring. They trained different image restoration models on several different datasets. In particular, they conducted experiments on the REalistic and Diverse Scenes (REDS) dataset [40], where images were not only blurred, but also degraded by compression. Their results surpassed the previous state-of-the-art on several benchmark datasets while using only a fraction of the computational resources.

In this work, we developed a novel weed segmentation model DeBlurWeedSeg to accurately segment weeds from sorghum and background in both sharp as well as motion blurred drone images. Therefore, we first conducted two consecutive UAV flights on the same agricultural field with different flight modes to capture sharp and motion blurred drone images and to enable an in-depth comparison of the effect of motion blur on weed segmentation. For this purpose, we trained a weed segmentation model using the easier to annotate sharp images (WeedSeg) and compared the segmentation behaviour of WeedSeg with a model combining NAFNet for deblurring with a subsequent segmentation step (DeBlurWeedSeg) on both, sharp and blurred images of a hold-out test-set. All data, containing blurry-sharp image pairs, as well as the corresponding expert generated semantic segmentation masks are published in our GitHub repository together with the code and the pre-trained segmentation models: https://github.com/grimmlab/DeBlurWeedSeg. The final model is available at Mendeley Data: https://data.mendeley.com/datasets/k4gvsjv4t3/1.

## Materials and methods

**Fig. 1:**
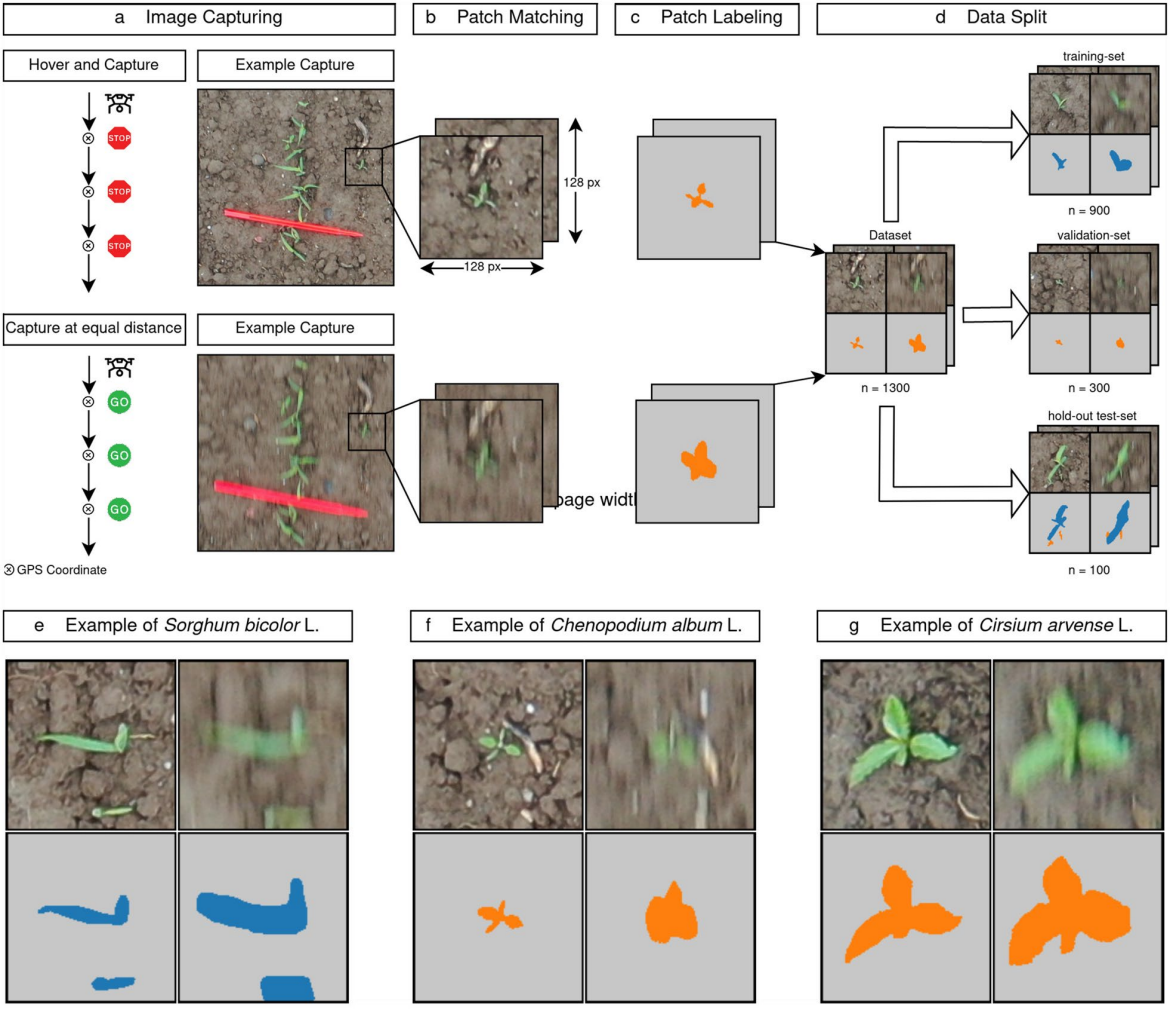
Overview of the image acquisition and data processing pipeline. **a** Image acquisition using two different drone settings. **b** Matching patches of the same image content. **c** Pixel-wise annotation of the patches. **d** Data splitting into training, validation and a hold-out test-set. **e–g** Example patches of sorghum and the main weeds

In the following, we first outline the image acquisition process, followed by the data preparation and processing pipeline used for this study. The main aspects of the data acquisition are summarized in Fig. 1a–d and explained in detail below. This description is followed by a detailed summary of our deblurring and weed segmentation models, including an overview of the hyperparameter optimization and evaluation metrics.

### Image acquisition

The images for this study were taken in an experimental agricultural sorghum field in southern Germany using a consumer-grade “DJI Mavic 2 Pro” drone equipped with a 20 MP Hasselblad camera (L1D-20c). The sorghum (*Sorghum bicolor* L.) crop was sown with a row spacing of 37.5 cm and a density of 25 seeds per $\text {m}^2$. The main weed species observed in this experimental field was *Chenopodium album* L. We also observed *Cirsium arvense* L. (Scop.) in small quantities (examples shown in Fig. 1e–g). We conducted automated drone missions at the end of September 2020, flying at an altitude of five meters with a drone velocity of 6.9 ${\text {km}}\,{\text{h}}^{-1}$ and an ambient wind current of 6 ${\text{km}}\, {\text{h}}^{-1}$. This resulted in a Ground Sampling Distance (GSD) of one millimeter, which is accurate enough to detect sorghum and weeds in early growth stages. Here, the sorghum was at growth stage 13 on the BBCH scale [41].

We used two different UAV settings for the flights, i.e. (i) “Hover and Capture” and (ii) “Capture at Equal Distance”, as shown in Fig. 1a. The first setting, “Hover and Capture” was used to stop and stabilize the UAV prior to image capture. This ensured sharp contours of the plants and mitigated the effects of motion blur. In addition, we repeated the flight on the same field and the same flight plan using the UAV’s “Capture at Equal Distance” setting. This caused the UAV to capture images at predetermined points without stopping and stabilizing. This resulted in degraded image quality with visible motion blur because the camera shutter was open while the UAV was moving. The images were captured with a shutter speed of $\frac{1}{120}s$, an aperture of approx. 4.0, an ISO of 100 and a manually added exposure bias of $-$ 0.3.

### Data processing

We first matched image pairs of sharp and motion blurred patches, as shown in Fig. 1b. This was necessary to ensure that each image pair contained the same or similar content, since flying with the “Capture at Equal Distance” setting resulted in images being captured at a slightly different location (difference of about 1 m) relative to the UAV’s flight direction due to GPS inaccuracies. In addition, several difficulties in the image capture process were identified, e.g. differences in the flight altitude which resulted in objects of different sizes, or that several plants were connected and appeared as a single plant in the blurred image due to the lower image quality. Therefore, a $128 \times 128$ $\text {px}^2$ patch was extracted for each plant instance, with the plant in the center, resulting in 1300 non-overlapping blurry-sharp image pairs as our final dataset. Further dataset statistics are summarized in Additional file 1.

Next, the dataset was manually semantically annotated using the open source software GIMP 2.10,^1^ as shown in Fig. 1c. This means that each image pair was separated into the three classes soil/background (gray), sorghum (blue), and weeds (orange).

For hyperparameter optimization and model selection, we sampled a distinct validation set. Therefore we split our dataset into three parts, as shown in Fig. 1d. The hold-out test-set for the final evaluation contains 100 image patches. From the remaining 1200 patches, we selected 25 % for the validation set. We stratified our dataset by the number of plants in each patch, as there was usually one plant instance present per patch. Additionally, we used the type of plants present in the patch (sorghum only, weeds only, both) as a second feature for stratification, since the majority of our dataset (about 70 %) consists of patches where only weeds are visible.

### Model selection

We implemented two models for the task of semantic weed segmentation for our comparison, namely WeedSeg and DeBlurWeedSeg. The WeedSeg model follows a classical encoder-decoder based architecture. It consists of an encoder part, where features of images are extracted and encoded into a high-level representation. This representation is of low spatial resolution, and therefore is decoded by a separate model to restore the shape of the input image. As decoder, we chose a UNet-based [14] architecture, similar to our previous work [12]. For the encoder, we use four different residual neural networks, namely ResNet-18, 34, 50, and 101. They were initialized with weights trained on the ImageNet dateset [42] to ensure comparability and faster training convergence.

**Fig. 2:**
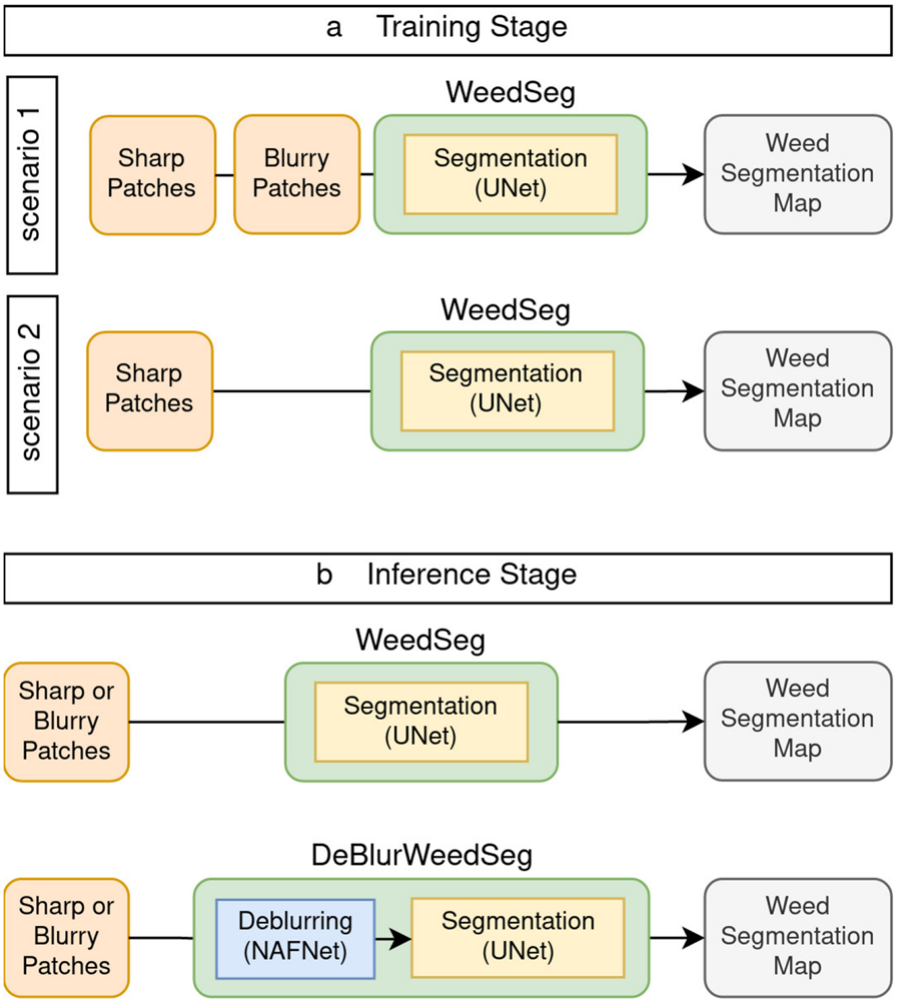
Our proposed weed segmentation model called DeBlurWeedSeg. The model consists of an additional deblurring model prior to weed segmentation model and is able to segment weeds in blurry and sharp images in the inference stage

In the training stage, we evaluated two different scenarios, as shown in Fig. 2a: First, similar to our previous model in [12], we used sharp and motion blurred images to train WeedSeg (Scenario 1). Therefore we collected sharp and motion blurred images together with their corresponding semantic ground-truths before training the model. In the second scenario we assumed, that only sharp image patches were available when training WeedSeg. This is more realistic, as generating high quality segmentation masks for motion blurred images is time-consuming and error-prone.

However, in a real-world scenario it cannot be assumed that the input images in the inference stage are all of the same quality (or distribution) as in the training stage (Fig. 2b). This can lead to a domain shift [43] and decrease prediction performance. In the case of weed segmentation using UAV captures, this shift could be caused by motion blur. Therefore, the classical WeedSeg model architecture may not generalize to motion blurred image captures. For this purpose, we propose DeBlurWeedSeg, a combined deblurring and segmentation model that can be used to detect weeds in both blurred and sharp image patches in production use. More importantly, the training phase is still performed only on sharp images. This has two advantages over WeedSeg: First, it eliminates the effort of semantically annotating motion-blurred UAV imagery, and second, training models on the new dataset is unnecessary, which might be resource intensive. DeBlurWeedSeg consists of two modules, a deblurring module based on the computationally efficient deblurring model NAFNet [39] and the segmentation model WeedSeg as described above.

### Model training and hyperparameter optimization

The performance of a model is highly sensitive to the hyperparameter configuration, especially when limited data are available. Therefore, hyperparameter optimization is a crucial step to select the best model. In this study, we used grid-search to optimize the learning rate and batch size for each encoder. For this purpose, ten different learning rates were selected from a log-uniform distribution starting from 1e−4 to 1e−3. The batch size was optimized starting from 128 up to the maximum possible size, depending on the size of the network architecture and the available GPU memory, in steps of 128. In total, we sampled 160 different hyperparameter sets for each scenario, as summarized in Table 1. Adam [44] was used as the optimizer with different learning rates. In addition, early stopping [45] was used to avoid overfitting.

**Table 1:** Summary of all hyperparameter sets used for each scenario

Encoder	Possible batch sizes	Num hyperparameter sets
ResNet-18	128, 256, 384, 512, 640, 768	60
ResNet-34	128, 256, 384, 512, 6 40	50
ResNet-50	128, 256, 384	30
ResNet-101	128, 256	20

### Evaluation metrics

Our proposed model DeBlurWeedSeg consists of two parts, as shown in Fig. 2. In the following, we summarize the metrics used to evaluate the deblurring and segmentation part of our model.

#### Deblurring metrics

To compare the output of a deblurring model, the quality of an image must be determined. Human evaluation is a reliable but expensive method. Alternatively, several metrics have been proposed for automatic Image Quality Assessment. The main goal is to imitate human perception with these metrics, which is a challenging task. We use the well-known Peak Signal-to-Noise Ratio (PSNR) [46] and Structural SIMilarity index (SSIM) [47] to evaluate the performance of the deblurring model. Although they are not suitable to measure the impact of artifacts that may have been introduced [48], they are still widely used in the literature.

Recent work [49] assessed the perceptual similarity between two images and evaluated different metrics. The authors concluded, that features on which deep learning-based networks are trained for classification tasks can be used to evaluate image quality. In this study, we use the metric called Learned Perceptual Image Patch Similarity (LPIPS) to evaluate our deblurring model.

#### Segmentation metrics

In supervised learning tasks, the confusion matrix is often used to evaluate the performance of different models. Considering a binary case, the classes are referred to as positive (P) and negative (N). A test example is defined as a true positive (TP), if it was correctly predicted to be positive. A true negative (TN) example is correctly predicted to be negative. Similarly, an example from the negative class that is misclassified as positive is called a false positive (FP), and a positive example that is misclassified as negative is called a false negative (FN). In the case of a multi-class classification problem, the values are calculated in a one-vs-all fashion. The confusion matrix is defined with a shape of NxN, where the N is the number of classes (three in our case). In addition, a set of quantitative metrics such as Accuracy (AC), Precision (PR), Recall (RE) and F1-Score (F1) can be derived from this matrix.

The weed segmentation task can be defined as classifying each pixel in an image. In our study, the dataset consists of three classes and we observed a high class imbalance especially for the majority class background (> 98% of pixels). This makes these evaluation metrics insufficient due to several reasons: Accuracy is dominated by the majority class. Precision does not provide insight into the number of samples from the FN. Also, Recall does not consider the number of samples from the FP. Additionally, a high F1-Value can be a result from the imbalance between PR and RE. To evaluate the segmentation performance of our models, we used the Sørensen-Dice coefficient [50], also called Dice-Score (DS). This function measures the similarity between two samples and is used in segmentation tasks with high class imbalance [51, 52]. It is mathematically defined as follows:

2 $$\begin{aligned} DS = \frac{2 \cdot TP}{2 \cdot TP + FP + FN}. \end{aligned}$$

We selected the best performing hyperparameter set with respect to this metric to train a final model with the combination of the training and validation set.

### Hardware and software

All models are implemented in Python 3.8.10 [53] using the packages numpy [54], pandas [55], pytorch [56], scikit-image [57], scikit-learn [58], albumentations [59] and kornia [60]. Our code is publicly available on GitHub.^2^ All experiments were conducted under Ubuntu 20.04 LTS on a machine with 104 CPU cores, 756 GB of memory, and four NVIDIA GeForce RTX 3090 GPUs. Each model was trained and evaluated on a single GPU.

## Results

In this section, we first give an overview of the training of WeedSeg and evaluate the deblurring model. Then, we evaluate the generalization performance of WeedSeg and compare it to DeBlurWeedSeg. We then analyze the predictions of both models in more detail. Finally, we compare the models qualitatively.

### WeedSeg model training and selection

To select the best performing WeedSeg model, we first trained ResNet-based feature extractors of different sizes using different hyperparameter sets. We show the best performing hyperparameter set for each feature extractor in Table 2. In summary, the ResNet-50 encoder performed best with a DS of 0.9048 using a batch size of 128. This model was selected for all further evaluations and comparisons. The results for all hyperparameter sets and their evaluations can be found in Additional file 2. The training curve on the validation set is shown in Additional file 3 .

**Table 2:** Best results on the validation set for each encoder and training strategy. The best performing combination is shown in bold

Scenario	Encoder name	Best of	Batch size	Step	Learning rate	DS $\uparrow$
1	ResNet-18	60	128	4900	$1.43 \cdot 10^{-4}$	0.8732
1	ResNet-34	50	384	4280	$2.37 \cdot 10^{-4}$	0.8655
1	ResNet-50	30	256	3540	$3.97 \cdot 10^{-4}$	0.8982
1	ResNet-101	20	256	4020	$5.40 \cdot 10^{-4}$	0.8995
2	ResNet-18	60	128	2160	$5.40 \cdot 10^{-4}$	0.8862
2	ResNet-34	50	384	1680	$5.11 \cdot 10^{-4}$	0.8837
**2**	**ResNet-50**	**30**	**128**	**2960**	${\textbf {5.40}} \cdot {\textbf {10}}^{{\textbf {-4}}}$	**0.9048**
2	ResNet-101	20	256	440	$7.35 \cdot 10^{-4}$	0.9011

### Deblurring evaluation

Next, the deblurring network (NAFNet) was evaluated on the hold-out test-set. The sharp image patches were considered as a reference. Here we can see, that SSIM and PSNR decreased slightly, as shown in Table 3. One possible reason could be, that these metrics do not correlate with human perception. Nevertheless, LPIPS was significantly reduced, indicating good deblurring performance.

**Table 3:** Evaluation of NAFNet on the hold-out test-set

	SSIM $\uparrow$	PSNR $\uparrow$	LPIPS $\downarrow$
Blurry	**0.39**	**19.61**	0.45
Deblurred	0.38	18.66	**0.32**

Dataset with the best performance is shown in bold

**Fig. 3:**
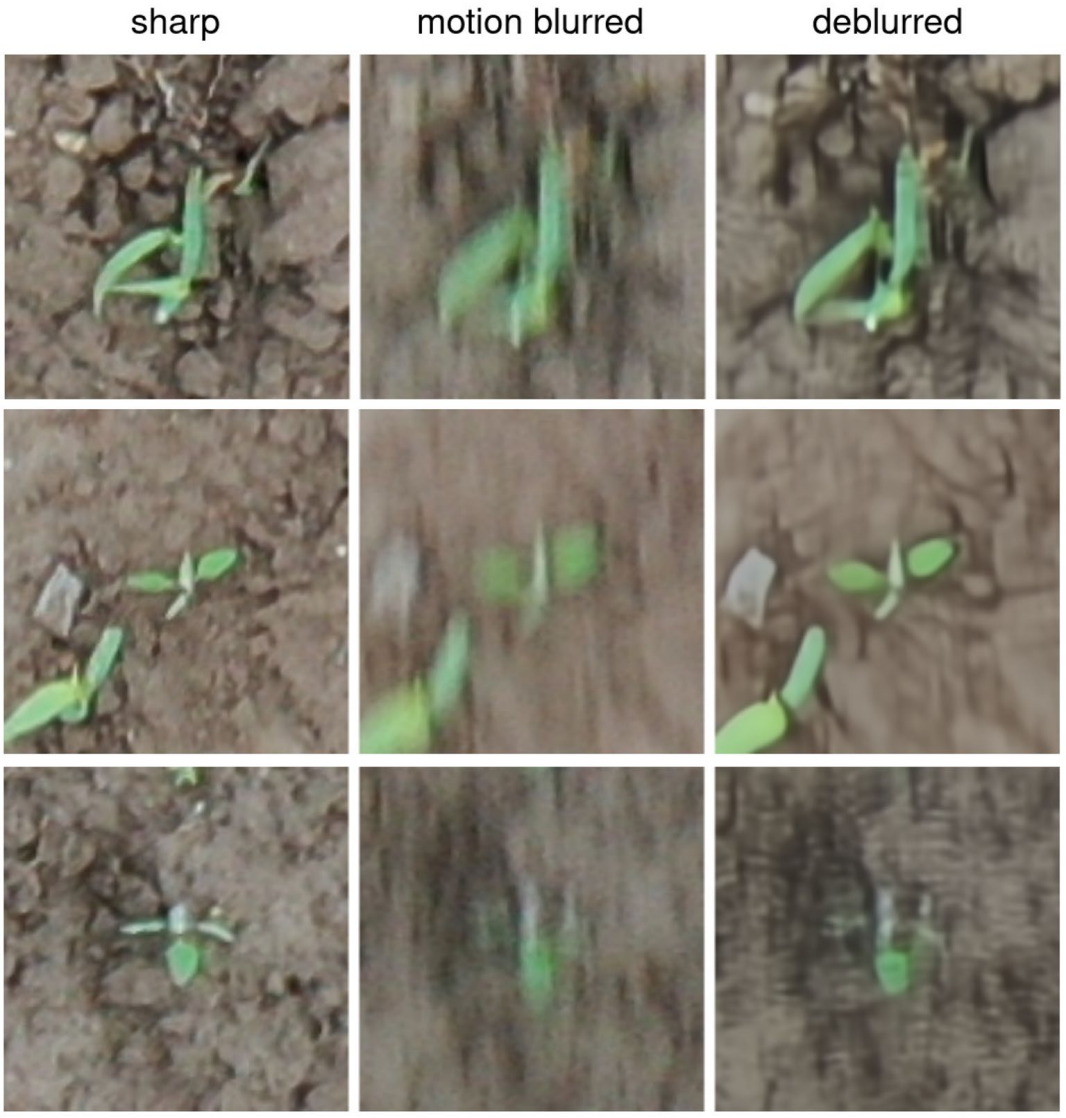
Qualitative examples of the deblurring step with NAFNet. This model was presented with motion blurred patches. Sharp patches only for reference

In addition, during a qualitative assessment of the deblurring step we observed a significant improvement in perceived sharpness, as shown in Fig. 3. Also, the deblurred patches showed less camera noise.

In some rare cases, the deblurring step failed for tiny weeds, making them indistinguishable (see Additional file 4). However, these patches were not critical to the average weed segmentation performance due to the tiny size of the plants.

### Generalization performance of WeedSeg and DeBlurWeedSeg

Next, we estimated the generalization performance of WeedSeg on a hold-out test-set. This set contains both blurred and sharp image patches, as shown in Table 4.

**Table 4:** DS for different scenarios and datasets. The sum of the sharp and motion blurred dataset is denoted in the column “Combined”

	Sharp	Motion blurred	Combined
WeedSeg (scenario 1)	0.8966	0.5864	0.7327
WeedSeg (scenario 2)	**0.9055**	0.5881	0.7381
DeBlurWeedSeg	0.8741	**0.8011**	**0.8373**

The best performing scenario is shown in bold

There were only little differences on our hold-out test-set based on the DS when trained on motion blurred and sharp images (scenario 1) or on sharp images only (scenario 2), as shown in Table 4. This is similar to the results on the validation-set (compare Table 2). Both models were able to segment sharp image patches with a high DS, but failed to segment image patches with motion blur. Therefore, we focus on scenario 2 in further analysis, as no motion blurred images and segmentation masks are needed during the training process. Our model DeBlurWeedSeg has a high DS on sharp and motion blurred images, resulting in a relative improvement of 13.4 % for the combined dataset. This is not surprising, since DeBlurWeedSeg contains a prior deblurring step and is thus able to sharpen blurred images before segmentation. Therefore, DeBlurWeedSeg is able to better generalize to new images with unknown drone settings.

**Fig. 4:**
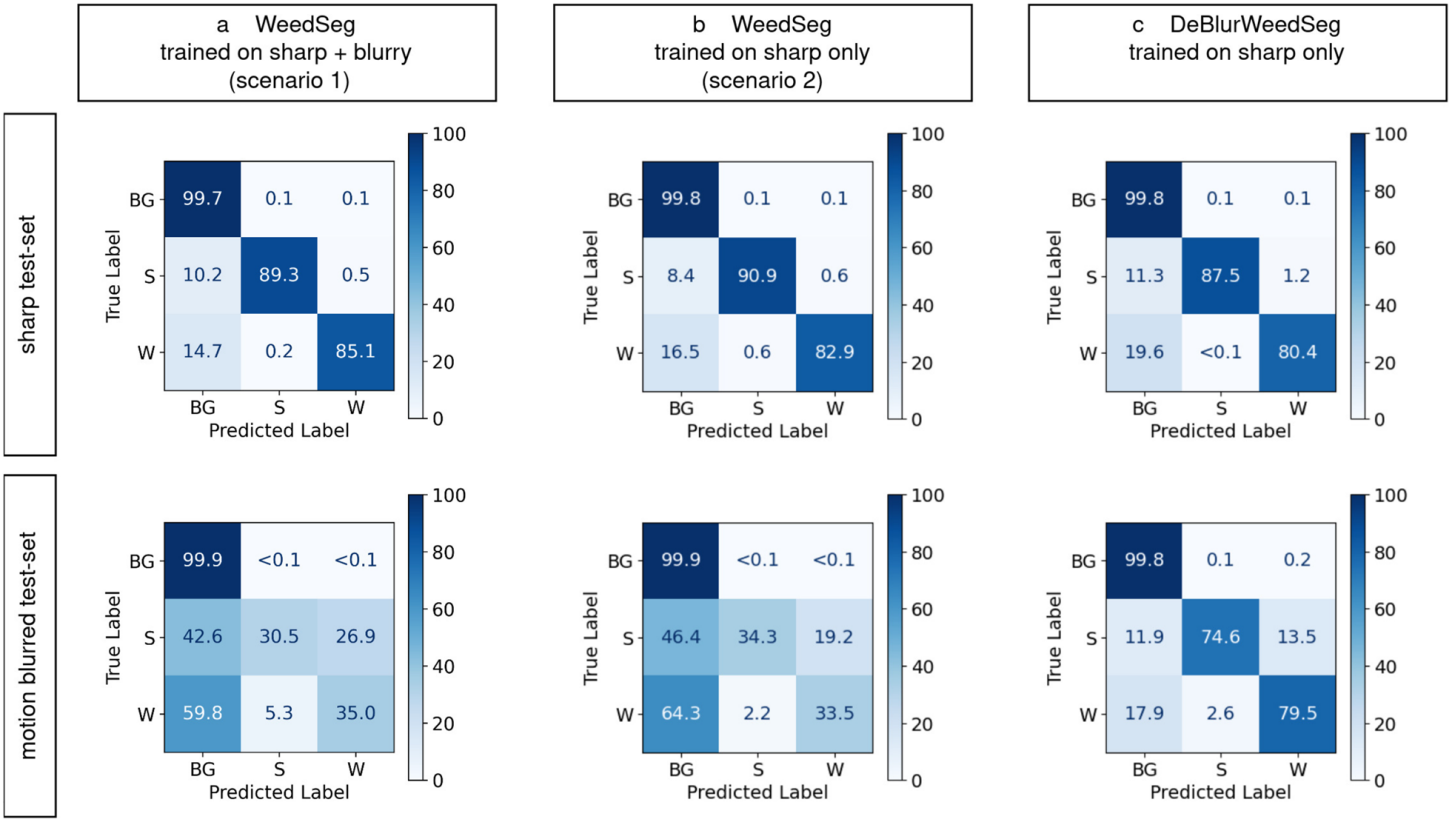
Pixel-based classification results shown in a normalized confusion matrix in percent. Background is denoted by BG, sorghum by S, and weeds by W. **a + b** The performance of WeedSeg is degraded by motion blurred image patches. This is true for training a segmentation model on sharp and blurry image patches (scenario 1) or on sharp parches only (scenario 2) **c** DeBlurWeedSeg has a significantly better performance on motion blurred image patches and is only slightly worse on sharp image patches

Furthermore, we provide a more detailed analysis of the hold-out test-set by showing a normalized confusion matrix of the accuracy calculated on a pixel basis (see Fig. 4). Here, the pixel-wise ground-truths and predictions were compared for each class, i.e., sorghum, weed and background.

We can see that the background class was predicted similarly well by all models, as indicated by an accuracy of more than 99.8 %. However, we clearly see a severe difference for sorghum and weed. We analyzed the segmentation capabilities for both sharp and motion blurred test images independently, as shown in Fig. 4. Here we can see that the performance of WeedSeg is highly accurate for sharp images. However, the performance drops severely for motion blurred images. This is to be expected in scenario2, since WeedSeg is trained only on sharp images. In particular, DeBlurWeedSeg performs well for both individual classes due to the prior deblurring step. We see a significant relative improvement in segmentation accuracy for blurred images of $\sim$ 117% for the class sorghum and $\sim$ 137% for the weed class. On sharp images, however, we observe a slight decrease in relative performance using DeBlurWeedSeg, i.e. 3.7% for sorghum and 3.1% for weed.

### Qualitative segmentation results

**Fig. 5:**
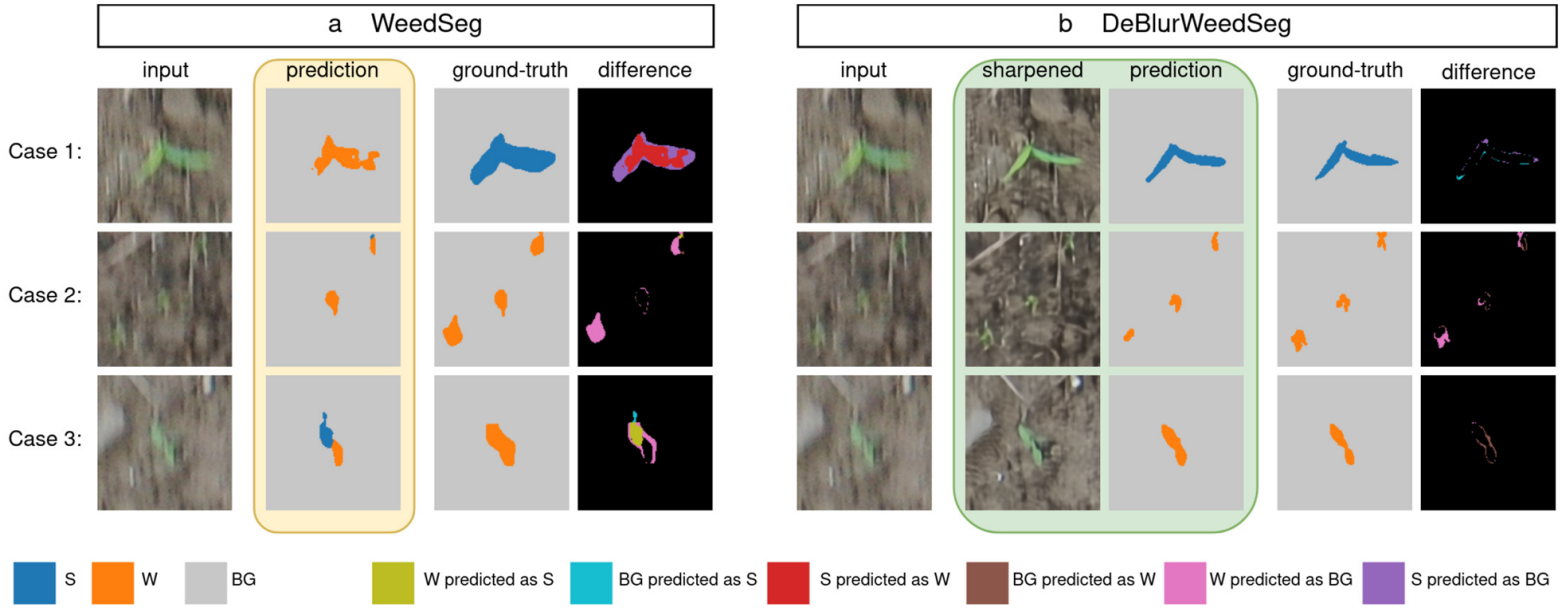
Examples of the blurred test-set. Background is gray, sorghum is blue, and weeds are orange. **a** Predictions from WeedSeg. **b** Predictions from DeBlurWeedSeg including the sharpened image patch

Finally, we analyze some example images from the test-set and their predictions in more detail. For this purpose, we generated segmentation difference maps of the prediction and the ground-truth and summarized them in Fig. 5.

For this analysis, we focus only on cases where WeedSeg predicted the motion blurred patches worse than the sharp counterpart. As shown in Fig. 5a, the failure cases of WeedSeg can be summarized in three cases: First, blurry sorghum plants were predicted as weeds (see Case 1). Second, small weeds could not be detected and were predicted as background (see Case 2). And third, parts of weed plants were misclassified as sorghum (see Case 3). In addition, we show the difference maps between ground-truth and prediction to highlight the areas of misclassification. All of these cases were successfully corrected by DeBlurWeedSeg, as shown in Fig. 5b. The remaining errors can be attributed to inaccuracies at the plant boundaries and tiny errors due to incorrect predictions of the entire plant instance, as shown in the difference map.

## Discussion

In this work, we trained a semantic segmentation model called WeedSeg, based on a UNet-shaped architecture with residual networks as encoders, to segment weeds from sorghum and background using only sharp training images. Selecting sharp images for training has the advantage that the annotation process is less time-consuming and error-prone compared to blurred and degraded images. Training and evaluating different segmentation models directly on blurred images has been studied extensively by Genze et al. in [12]. In the current study study, we aimed to investigate the generalization abilities of models trained under idealized conditions and then deployed in productive environments. We observed a significant drop in performance when applying WeedSeg to naturally motion blurred images, i.e. motion blurred images due to non-ideal flight settings. Also, training a model on sharp and motion blurred image patches (scenario 1) yielded inferior results. We identified motion blur as a major bottleneck for semantic weed segmentation. Therefore, we generated a dataset containing matching blurry-sharp image pairs of sorghum and weed plants and their corresponding semantic ground-truths.

In this study, we proposed a combined deblurring and semantic segmentation model DeBlurWeedSeg that is able to segment sorghum and weeds from the background in sharp and motion blurred images. Here, NAFNet [39], a computationally efficient deblurring model is used as a prior step to produce a sharpened version from the blurred input images, which is then segmented by our weed segmentation model. Our proposed model achieves significantly better performance with motion blurred and sharp image patches. Nevertheless, DeBlurWeedSeg still misclassified 13.5% of the sorghum pixels as weeds in motion blurred patches (see Fig. 4c), indicating that there is room for improvement in classifying the correct plant species. These errors could be resolved by training the weed segmentation model with additional sorghum images, as our dataset contains more images of weeds. Also, there was a slight drop in performance when using DeBlurWeedSeg on sharp image patches. This might be an indication, that the segmentation model is slightly dependent on low-level noise that is present in the sharp image patches (i.e. ISO noise) and is subject of a future study.

Although DeBlurWeedSeg performed well on our hold-out test-set, there are a number of factors that were not evaluated in this study. First, our dataset was generated from a single UAV mission over a specific agricultural field and the sorghum plants were at a low growth stage of BBCH 13. However, the weed flora may be different in other regions and for different growth stages of sorghum. Second, different weather conditions and sampling times could affect the illumination of the images and thus the segmentation performance. Here, we focused on one UAV flight where *Chenopodium album* L. was the main weed present in the field. As future work, we would like to evaluate this method on a variety of growth stages and weed species.

This research could also be integrated into agricultural robots to deal with motion blur on the fly, which is the subject of another study.

## Conclusion

Accurate detection and segmentation of weeds in the early growth stages of sorghum is critical for effective weed management. However, UAVs are prone to motion blur, which is a major problem for real-world applications and use of deep-learning based weed segmentation models. In this study, we propose a combined deblurring and weed segmentation model DeBlurWeedSeg. We demonstrate that we can efficiently mitigate the performance loss that was caused by motion blur. In addition, this method could be used to segment already sharp image patches without a substantial drop in performance. Finally, this model could lead to better weed control due to lower error rates in weed detection and help to enforce agricultural robots in combination with mechanical weed control.

### Supplementary Information

**Additional file 1.** Dataset Statistics.

**Additional file 2.** Results of hyperparameter optimisation.

**Additional file 3.** Training Curve of the best performing hyperparameter set.

**Additional file 4.** Insufficient cases of the deblurring step.

## Data Availability

All data, annotations and code is publicly available on GitHub under https://github.com/grimmlab/DeBlurWeedSeg and Mendeley Data under https://data.mendeley.com/datasets/k4gvsjv4t3/1.

## Abbreviations

CNN: Convolutional neural network
GSD: Ground sampling distance
UAV: Unmanned aerial vehicle
PSNR: Peak signal to noise ratio
SSIM: Structural Similarity Index
LPIPS: Learned perceptual image patch similarity

## Footnotes

1 https://www.gimp.org.

2 https://github.com/grimmlab/DeBlurWeedSeg.
