# Supplementary figures and images for: Improved weed segmentation in UAV imagery of sorghum fields with a combined deblurring segmentation model
Source: Plant Methods. 2023 Aug 22;19:87. doi: 10.1186/s13007-023-01060-8 (PMC10463442; doi:10.1186/s13007-023-01060-8)

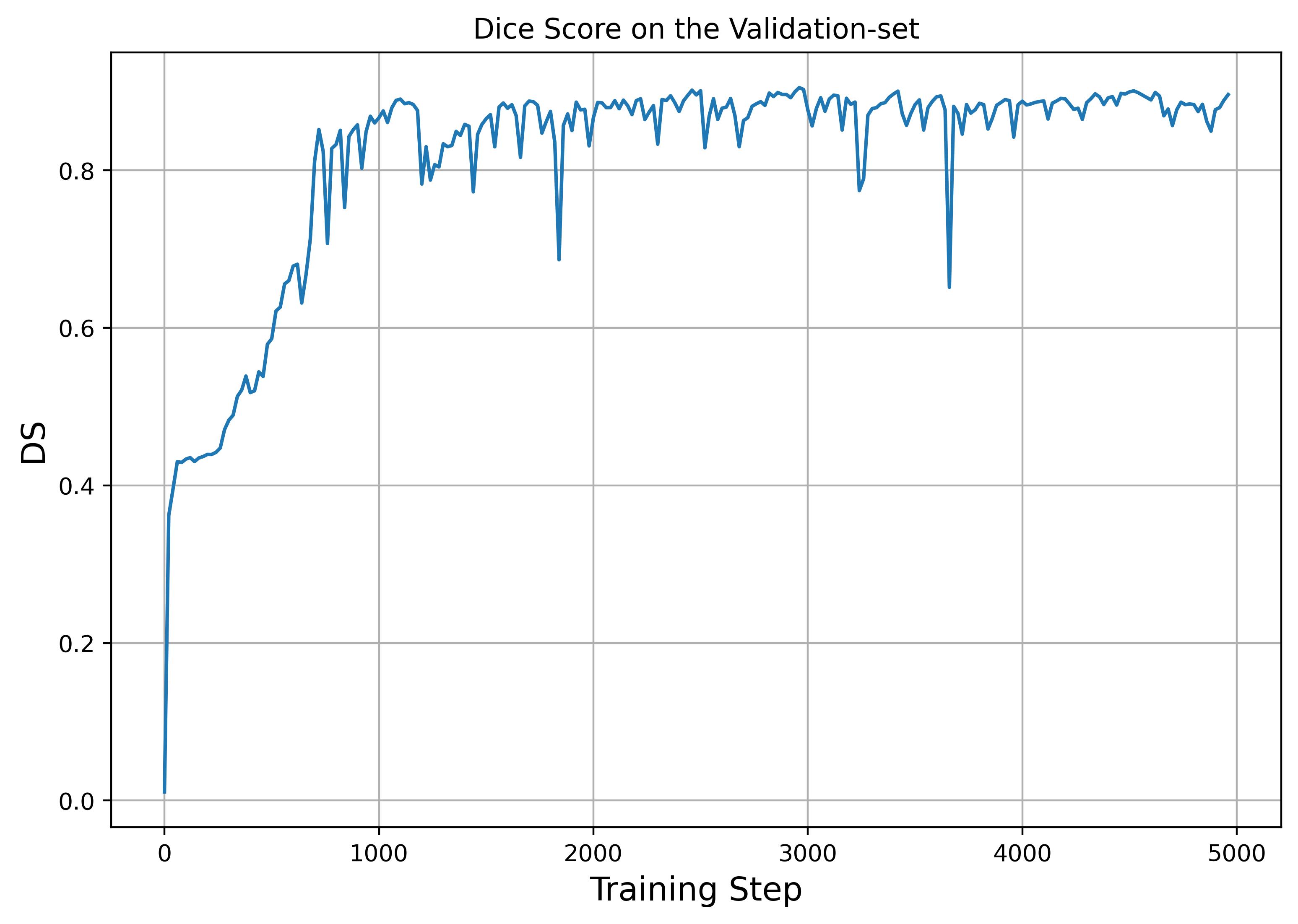

Supplement: Supplementary file 3 — Additional file 3. Training Curve of the best performing hyperparameter set. [file 13007_2023_1060_MOESM3_ESM.jpg]
