# Supplementary material for: Improved weed segmentation in UAV imagery of sorghum fields with a combined deblurring segmentation model
Source: Plant Methods. 2023 Aug 22;19:87. doi: 10.1186/s13007-023-01060-8 (PMC10463442; doi:10.1186/s13007-023-01060-8)
Supplement: Supplementary file 4 — Additional file 4. Insufficient cases of the deblurring step. [file 13007_2023_1060_MOESM4_ESM.pdf]

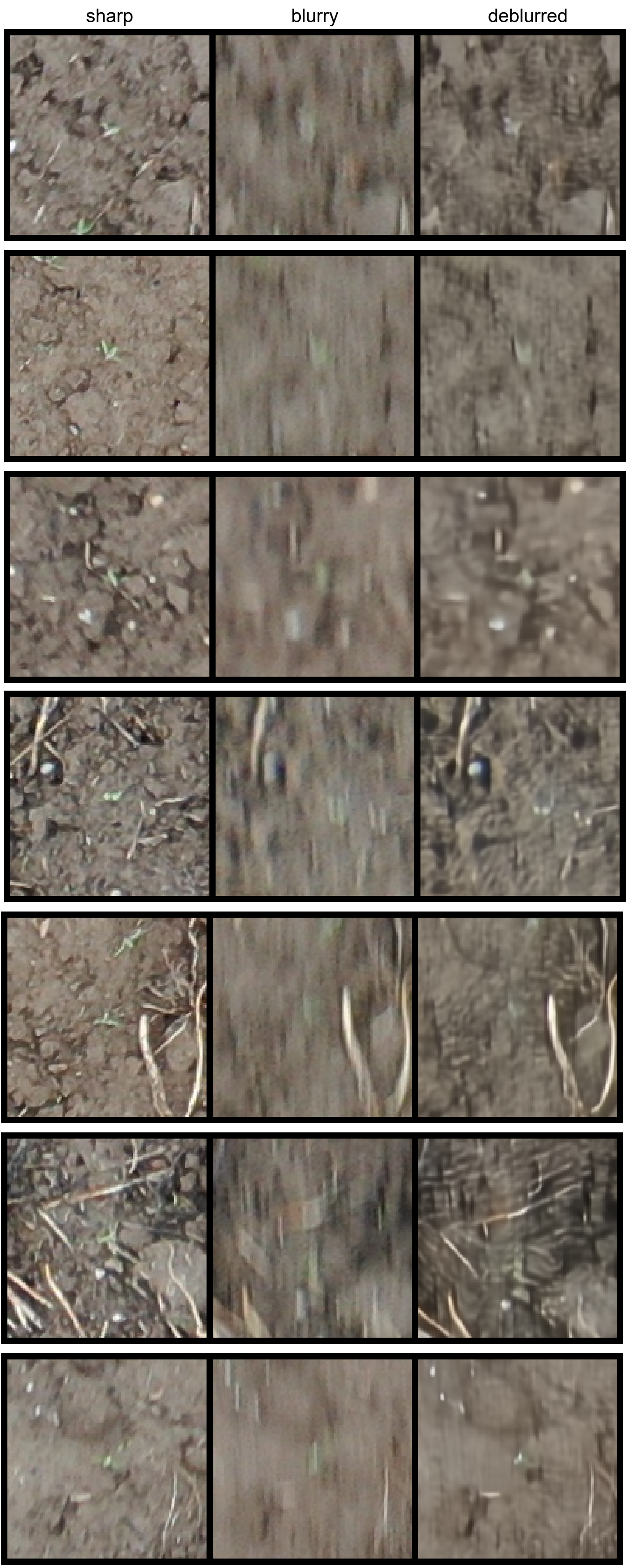

Insufficient results by the deblurring step of our DeBlurWeedSeg model. The weed in the deblurred patches can hardly be recognised. The model was presented with the blurry image patch and predicted the deblurred version. Sharp image patch for reference.
